# Utility of established prognostic scores in COVID-19 hospital admissions: multicentre prospective evaluation of CURB-65, NEWS2 and qSOFA

**DOI:** 10.1136/bmjresp-2020-000729

**Published:** 2020-12-07

**Authors:** Patrick Bradley, Freddy Frost, Kukatharmini Tharmaratnam, Daniel G Wootton, NW Collaborative Organisation for Respiratory Research, Mahin Ahmad

**Affiliations:** 1 Department of Respiratory Medicine, Blackpool Teaching Hospitals NHS Foundation Trust, Blackpool, UK; 2 Department of Respiratory Medicine, Liverpool University Hospitals NHS Foundation Trust, Liverpool, UK; 3 Institute of Infection, Veterinary and Ecological Sciences, University of Liverpool, Liverpool, UK; 4 Department of Health Data Science, University of Liverpool, Liverpool, UK

**Keywords:** viral infection, pneumonia, respiratory infection

## Abstract

**Introduction:**

The COVID-19 pandemic is ongoing, yet, due to the lack of a COVID-19-specific tool, clinicians must use pre-existing illness severity scores for initial prognostication. However, the validity of such scores in COVID-19 is unknown.

**Methods:**

The North West Collaborative Organisation for Respiratory Research performed a multicentre prospective evaluation of adult patients admitted to the hospital with confirmed COVID-19 during a 2-week period in April 2020. Clinical variables measured as part of usual care at presentation to the hospital were recorded, including the Confusion, Urea, Respiratory Rate, Blood Pressure and Age Above or Below 65 Years (CURB-65), National Early Warning Score 2 (NEWS2) and Quick Sequential (Sepsis-Related) Organ Failure Assessment (qSOFA) scores. The primary outcome of interest was 30-day mortality.

**Results:**

Data were collected for 830 people with COVID-19 admitted across seven hospitals. By 30 days, a total of 300 (36.1%) had died and 142 (17.1%) had been in the intensive care unit. All scores underestimated mortality compared with pre-COVID-19 cohorts, and overall prognostic performance was generally poor. Among the ‘low-risk’ categories (CURB-65 score<2, NEWS2<5 and qSOFA score<2), 30-day mortality was 16.7%, 32.9% and 21.4%, respectively. NEWS2≥5 had a negative predictive value of 98% for early mortality. Multivariable logistic regression identified features of respiratory compromise rather than circulatory collapse as most relevant prognostic variables.

**Conclusion:**

In the setting of COVID-19, existing prognostic scores underestimated risk. The design of new prognostic tools should focus on features of respiratory compromise rather than circulatory collapse. We provide a baseline set of variables which are relevant to COVID-19 outcomes and may be used as a basis for developing a bespoke COVID-19 prognostication tool.

## Introduction

The novel coronavirus SARS-CoV-2 is causing a global pandemic of the infectious disease termed COVID-19. COVID-19 is frequently associated with a pneumonia syndrome and the large ISARIC (International severe acute respiratory and emerging infection consortium) observational study estimates a case fatality rate of 33% among those admitted to hospital.^1^ Prognostic scores can improve clinical decision making, and pre-COVID-19 several scores had been extensively validated and supported by national and international guidelines for application in the context of acute infectious disease.^2–4^

The Confusion, Urea, Respiratory Rate, Blood Pressure and Age Above or Below 65 Years (CURB-65) score is a community-acquired pneumonia (CAP)-specific tool for predicting all-cause mortality within 30 days. CURB-65 has been validated across large, diverse patient populations and has been endorsed by national and international guidelines as an aid to clinical decision making.^5–9^ The National Early Warning Score 2 (NEWS2) is a scoring system based on routine physiological measurements, and its implementation into all English National Health Service (NHS) hospitals has beenbmandated in the pre-COVID-19 era.^10^ NEWS2 is a disease agnostic early warning tool used to trigger escalation of care in the deteriorating patient, with high scores being associated with death or unanticipated intensive care unit (ICU) admission within 24 hours.^2^ The Quick Sequential (Sepsis-Related) Organ Failure Assessment (qSOFA) score is a tool for predicting mortality and ICU admission among patients with suspected infection in prehospital, emergency department and ward settings. It has been validated through large datasets, and has gained prominence following its recommendation by the Sepsis-3 task force.^4 11^

At the onset of the UK epidemic, in the absence of COVID-19-specific prognostic tools, CURB-65, NEWS2 and qSOFA remained in widespread use, but little was known about their validity in the COVID-19 setting. The primary aim of this study was to determine the performance characteristics of these scores in the context of COVID-19 and, secondarily, to investigate potential components of a COVID-19-specific prognostication tool for future validation.

## Methods

### Study setting and participants

The North West Collaborative Organisation for Respiratory Research (NW-CORR) collected data during the 2-week period from 1 April 2020 to 14 April 2020 on prospective adult COVID-19 admissions at seven acute hospitals in North West England. NW-CORR constitutes a group of research-interested, respiratory, specialist trainee grade doctors, and the recruiting centres were those with an NW-CORR member available. Collaborators were asked to record routinely collected clinical data for consecutive patients admitted to their hospitals who met the Public Health England inpatient case definition for COVID-19^12^ and had a positive SARS-CoV-2 PCR test. There were no exclusion criteria. No approach to the patient was made, and only fully anonymised, routinely available clinical information was collated; on this basis, consent was not required under pandemic-specific guidance from the NHS Human Research Authority (https://www.hra.nhs.uk/covid-19-research/guidance-using-patient-data/).^13^

### Patient and public involvement

Healthcare professionals with COVID-19 were involved in the design, conception and conduct of the study. All agreed the project was desirable and non-intrusive for patients and deliverable during a time of crisis.

### Outcomes and prognostic scores

Data collected included demographic characteristics, vital signs and blood test results. All physiological values and blood results constituting components of the CURB-65, NEWS2 and qSOFA scores were the earliest measurement recorded in the hospital. The variables included in each risk score are shown in table 1. At the point of data entry, collaborators were also asked to comment on the presence or absence of consolidation on chest radiography. Outcomes studied were 30-day all-cause mortality, 72-hour mortality and ICU admission. With respect to the three risk scores, these outcome measures are in some cases validated and in other cases unvalidated but widely applied in clinical practice; in the unvalidated context, the analysis was therefore exploratory.

**Table 1:** Components of the NEWS2, qSOFA and CURB-65 scores

CURB-65	NEWS2	qSOFA
Confusion Urea>7 mmol/L Respiratory rate: 30 breaths/min BP: systolic<90 or diastolic 60 mm Hg Age 65 years	Respiratory rate SpO_2_ Supplemental O_2_ use Heart rate Altered consciousness Temperature	Altered mental status Respiratory rate: 22 breaths/min BP: systolic 100 mm Hg

CURB-65, Confusion, Urea, Respiratory Rate, Blood Pressure and Age Above or Below 65 Years; NEWS2, National Early Warning Score 2; qSOFA, Quick Sequential (Sepsis-Related) Organ Failure Assessment.

### Data handling

**Figure 1:**
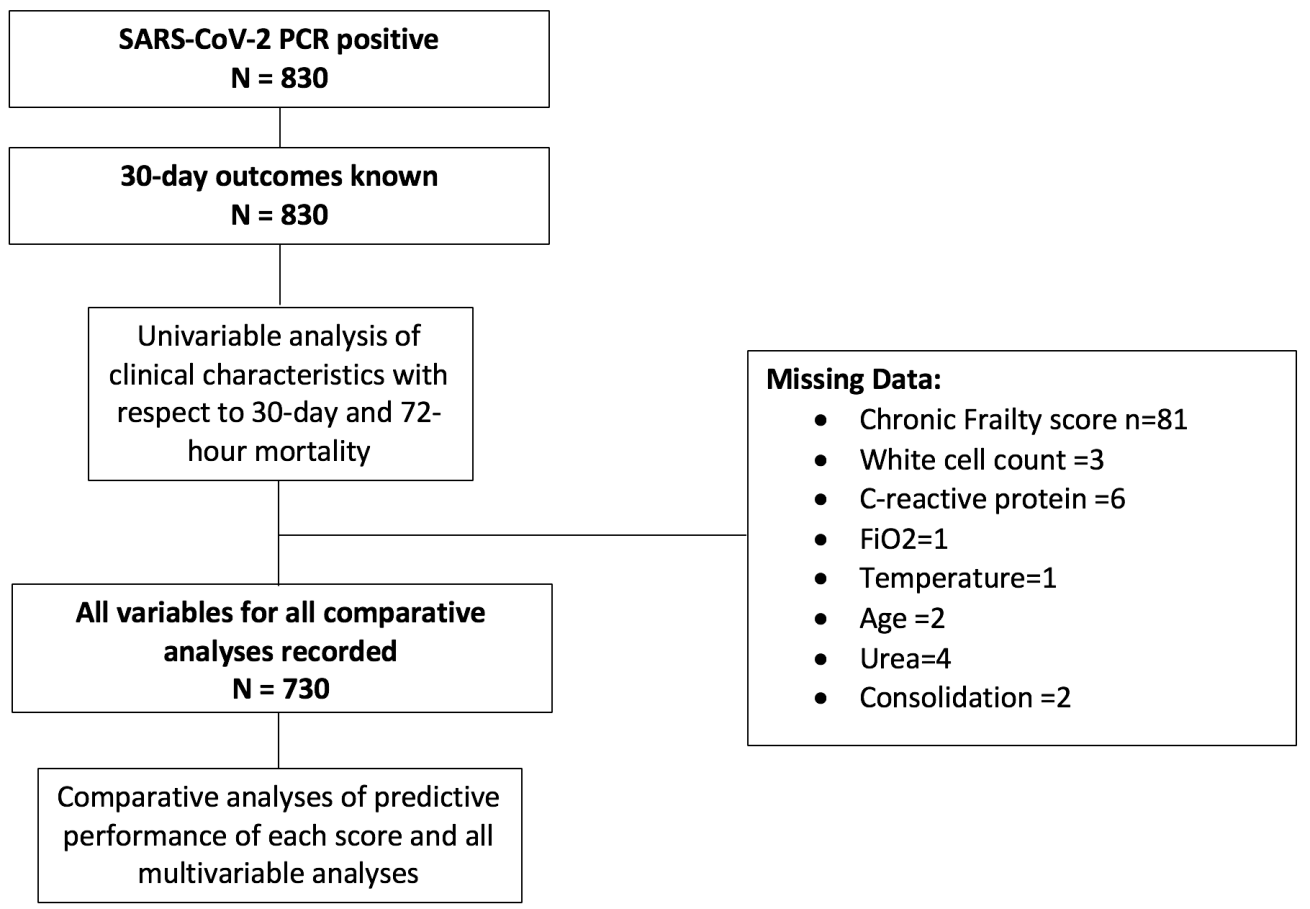
Patient flowchart describing cohort for each analysis and missing data. Analysis of CURB-65 scores was restricted to patients with consolidation on chest radiograph. CURB-65, Confusion, Urea, Respiratory Rate, Blood Pressure and Age Above or Below 65 Years; FiO_2_, fraction of inspired oxygen.

Anonymised study data were collated centrally and managed using the secure, web-based software platform Research Electronic Data Capture (Vanderbilt University, USA) hosted at the University of Liverpool. In general, there were minimal missing data with >99% completeness for all constituent variables of the three prognostic scores (table 2 and figure 1).

**Table 2:** Baseline demographics and clinical characteristics with comparison by outcome at 30 days

	Missing	All	Death by 30 days		
			Alive	Dead	P value
n		830	530	300	
Characteristics at presentation					
Age (years)	1 (0.1)	70 (58–80)	65 (55–76)	76 (67–85)	<0.001
Sex (male)	0 (0.0)	509 (61.3)	309 (58.3)	200 (66.7)	0.02
CFS score	81 (9.8)	4 (2–6)	3 (2–5)	5 (3–6)	<0.001
Temperature (°C)	3 (0.4)	37.5 (36.8–38.2)	37.4 (36.8–38.1)	37.6 (36.8–38.4)	0.15
Respiratory rate (breaths/min)	2 (0.2)	24 (20–28)	22 (20–26)	24 (21–30)	<0.001
Heart rate (beats/min)	1 (0.1)	94 (82,108)	95 (82–108)	91 (80–110)	0.28
Systolic BP (mm Hg)	2 (0.2)	130 (115–145)	130 (117–145)	131 (113–145)	0.78
Diastolic BP (mm Hg)	2 (0.2)	75.0 (65.0–84.0)	76.0 (67.0–85.0)	72 (63–81)	<0.001
SpO_2_	1 (0.1)	94 (90–96)	94 (91–96)	93 (89–96)	0.002
Supplemental oxygen (%)	2 (0.2)	340 (41.0)	183 (34.5)	157 (52.3)	<0.001
SpO_2_:FiO_2_ ratio	2 (0.2)	414 (283 to 448)	429 (339 to 452)	354 (189 to 443)	<0.001
Confusion	0 (0.0)	183 (22.0)	90 (17.0)	93 (31.0)	<0.001
Investigations					
Urea (mmol/L)	8 (1.0)	7.2 (4.9–11.1)	6.3 (4.6–9.2)	9.8 (7.0–15.1)	<0.001
Consolidation	4 (0.5)	684 (82.8)	425 (80.5)	259 (86.9)	0.02
CRP (mg/L)	10 (1.2)	104.0 (47.8–173.1)	92.0 (42.5–158.0)	117.0 (59.0–195.0)	<0.001
WCC (10^9^/L)	4 (0.5)	7.4 (5.6–10.1)	7.3 (5.5–9.8)	7.6 (5.7–10.6)	0.20
Neutrophils (10^9^/L)	7 (0.8)	5.8 (3.9–8.55)	5.6 (3.8–7.9)	5.9 (4.0–9.1)	0.26
Lymphocytes (10^9^/L)	9 (1.1)	0.8 (0.6–1.1)	0.9 (0.6–1.2)	0.7 (0.5–1.0)	<0.001
NLR	9 (1.1)	154.0 (91.0–201.0)	155.0 (95.5–201.0)	152.5 (85.3–201.0)	0.62

P values are calculated using Fisher’s exact test for categorical variables, Mann-Whitney test for continuous variables since none of the continuous variables follow the assumption of normality (Shapiro normality test p value of ≤0.001 for all of them)

Data are presented as n (%) or median (IQR).

BP, blood pressure; CFS, Clinical Frailty Scale; CRP, C reactive protein; FiO_2_, fraction of inspired oxygen; NLR, neutrophil:lymphocyte ratio; SpO_2_, oxygen saturation by pulse oximetry; WCC, white cell count.

### Statistical analysis

Scores were assessed individually against their validated outcomes and overall for their ability to identify people at risk of mortality within 72 hours (early death) and 30 days of admission. This analysis included sensitivity and specificity of each score’s respective risk strata followed by an evaluation of discrimination and calibration in keeping with TRIPOD guidelines.^14^ Discriminatory ability was assessed by comparison of the corresponding receiver operating characteristic curves with computation of area under the curve (AUC). Calibration was assessed visually by plotting the observed risk for a score’s individual strata against published reference risk derived from their original validation. In order to allow direct comparison of the clinical scoring systems, only patients with complete data for all variables were included in comparative statistical analyses. Data for the clinical parameter Clinical Frailty Scale (CFS) score was missing from one centre where this data was not recorded; since this was confined to one centre and therefore was not randomly missing, analyses were restricted the population where all variables were available.

Multiple logistic regression models were fitted for each of the outcome variables (30-day mortality, 72-hour mortality and ICU admission) using each score (CURB-65, NEWS2 and qSOFA). With the aim of identifying variables relevant to COVID-19 outcomes and in order to assess the association of each individual variable (eg, age and respiratory rate) with each of the outcomes, multiple regression modelling using all variables was fitted by applying backward variable selection. Data heterogeneity introduced by differences among hospitals was assessed by adding a random intercept in the model. However, clustering by hospital did not improve the accuracy of the model, and the final models did not include a random term. The performance of the fitted models was assessed by sensitivity, specificity, positive predictive value (PPV), negative predictive value (NPV) and AUC. These analyses were performed using the statistical software package pROC in R V.3.5.3. pROC features internal cross validation based on bootstrap sampling method.

## Results

Data were collected and recorded for 830 patients admitted to seven hospitals in the North West of England, encompassing both secondary and tertiary care hospitals (see online supplemental material). Clinical characteristics and observations at admission are presented in table 2. Overall, 509/830 (61.3%) were male with a median age of 70 years (IQR 58–80) and a Rockwood CFS score of 4 (IQR 2–6). Within 72 hours of admission, 63/830 (7.6%) patients had died and 125/830 (15.0%) had been admitted to ICU. At 30 days, 300/830 (36.1%) had died; 452/830 (54.5%) has been discharged; and 78/830 (9.4%) remained in the hospital. During the 30-day period, 142 (17.1%) were admitted to critical care, of whom 65 (45.8%) died. A comparison of clinical characteristics based on 30-day and 72-hour mortality is presented in table 2 and online supplemental table S1, respectively.

10.1136/bmjresp-2020-000729.supp1 Supplementary data

**Figure 2:**
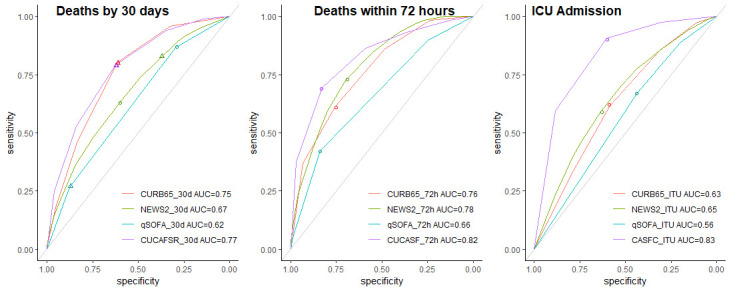
Receiver operating characteristic plots for death within 72 hours and ICU admission based on the models defined as the sum of the corresponding predictors. Circles denote the sensitivity and specificity achieved by the optimal threshold from fitted models. AUC, area under the curve; CUCAF-SR, Clinical Frailty Scale, Urea, Consolidation, Age, FiO_2_, Sex, Respiratory rate; CUCA-SF, Clinical Frailty Scale, Urea, Consolidation, Age, SpO_2_, FiO_2_; CURB-65, Confusion, Urea, Respiratory Rate, Blood Pressure and Age Above or Below 65 Years; ICU, intensive care unit; NEWS2, National Early Warning Score 2; qSOFA, Quick Sequential (Sepsis-Related) Organ Failure Assessment.

**Figure 3:**
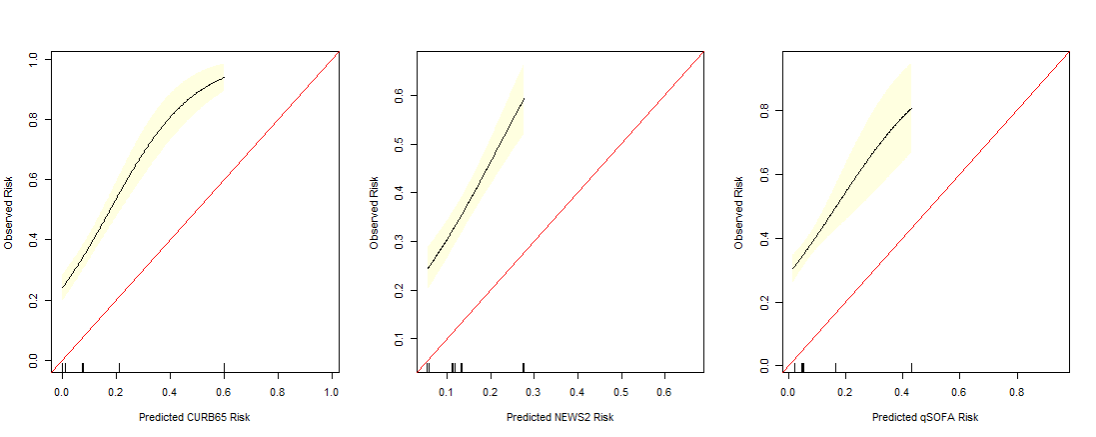
Calibration plots of the predicted risk of 30 day mortality (based on published validation studies) for CURB65,^15^ NEWS2^10^ and qSOFA^16^ against observed risk in COVID-19 hospital admissions. CURB-65, Confusion, Urea, Respiratory Rate, Blood Pressure and Age Above or Below 65 Years; NEWS2, National Early Warning Score 2; qSOFA, Quick Sequential (Sepsis-Related) Organ Failure Assessment.

The discriminatory ability of each score was assessed for death within 30 days, death within 72 hours and admission to critical care, and are presented in figure 2. In general, performance was modest, with AUCs ranging from 0.62 to 0.77. Calibration was computed by comparing the predicted risk from each score against the respective observed risk in the study cohort. Visual comparison of each calibration plot confirmed slopes of >1 and intercepts of >0, suggestive of underestimation (see figure 3).

To test scores’ performance at their individual validated thresholds, the sensitivity, specificity, NPV and PPV were calculated for patients with complete datasets (see figure 1) and are presented in table 3. Overall, for 30-day mortality, scores failed to accurately identify a low-risk group, with mortality in the lowest-risk strata ranging from 16% to 33%. For 72-hour mortality, a CURB-65 threshold of 2 and A NEWS2 threshold of 5 both identified a low-risk group with just 2-3% mortality and NEWS2 achieved a sensitivity of 92% with an NPV of 98%. All scores performed poorly in predicting admission to ICU (see online supplemental table S2). The relative likelihood of mortality at each stratum of each score is presented in table 4.

**Table 3:** Diagnostic performance of individual scores for 30-day and 72-hour mortality

	Score (n)	Death (%)	Sensitivity	Specificity	PPV	NPV
Death by 30 days						
CURB-65 (n=605)*	<2 (273)	46 (16.8)	0.80	0.61	0.57	0.83
	≥2 (332)	188 (56.6)				
	<3 (434)	125 (28.8)	0.47	0.83	0.64	0.71
	≥3 (171)	109 (63.7)				
NEWS2 (n=730)	<5 (215)	46 (21.4)	0.83	0.37	0.43	0.79
	≥5 (515)	224 (43.5)				
qSOFA (n=730)	<2 (596)	196 (32.9)	0.27	0.87	0.55	0.67
	≥2 (134)	74 (55.2)				
Death within 72 hours						
CURB-65 (n=605)	<2 (273)	8 (2.9)	0.86	0.48	0.15	0.97
	≥2 (332)	49 (14.8)				
	<3 (434)	22 (5.1)	0.61	0.75	0.20	0.95
	≥3 (171)	35 (20.5)				
NEWS2 (n=730)	<5 (215)	5 (2.3)	0.92	0.31	0.10	0.98
	≥5 (515)	54 (10.5)				
qSOFA (n=730)	<2 (596)	34 (5.7)	0.42	0.84	0.19	0.94
	≥2 (134)	25 (18.7)				

*Analysis restricted to those with consolidation on chest radiograph.

CURB-65, Confusion, Urea, Respiratory Rate, Blood Pressure and Age Above or Below 65 Years; NEWS2, National Early Warning Score 2; NPV, negative predictive value; PPV, positive predictive value.

**Table 4:** OR of death within 30 days and 72 hours for the individual strata of each score

Model	Score	OR (95% CI)	P value
30-day mortality			
CURB-65	0 (reference)		
	1	4.04 (1.91 to 8.53)	<0.001
	2	11.56 (5.65 to 23.64)	<0.001
	3	17.87 (8.46 to 37.73)	<0.001
	≥4	29.65 (12.59 to 69.82)	<0.001
NEWS2	<5 (reference)		
	≥5	2.83 (1.95 to 4.09)	<0.001
qSOFA	<2 (reference)		
	≥2	2.52 (1.72 to 3.68)	<0.001
72h mortality			
CURB-65	0 (reference)		
	1	6.64 (0.81 to 54.71)	0.079
	2	12.29 (1.59 to 94.71)	0.016
	3	18.43 (2.38 to 142.52)	0.005
	≥4	71.29 (9.29 to 547.34)	<0.001
NEWS2	<5 (reference)		
	≥5	4.92 (1.94 to 12.48)	0.001
qSOFA	<2 (reference)		
	≥2	3.79 (2.18 to 6.61)	<0.001

CURB-65, Confusion, Urea, Respiratory Rate, Blood Pressure and Age Above or Below 65 Years; qSOFA, Quick Sequential (Sepsis-Related) Organ Failure Assessment.

When all individual variables were considered, multivariable logistic regression revealed that confusion and blood pressure (BP) were less relevant to 30-day mortality than urea, respiratory rate and age when computed as part of the CURB-65 score (see online supplemental table S3). In a similar fashion, the most severe BPs and HR strata in NEWS2 (score of 3: systolic BP≤90 mm Hg, HR>130 or <41/min) were not independently associated with poorer outcomes in the NEWS2 model (see online supplemental table S4), whereas the corresponding fraction of inspired oxygen (FiO_2_) and respiratory rate strata were relevant to mortality (OR 2.00 (1.4–2.8), p<0.001 and OR 1.9 (1.3–2.9), p=0.003, respectively).

Finally, a backward selection multivariable model fitted for each outcome identified de novo a set of variables independently associated with 30-day mortality (Clinical Frailty Scale, Urea, Consolidation, Age, FiO_2_, Sex, Respiratory rate (CUCAF-SR)) and a similar set of variables for 72-hour mortality (Clinical Frailty Scale, Urea, Consolidation, Age, SpO_2_, FiO_2_ (CUCA-SF)), as shown in table 5.

**Table 5:** Important prognostic variables identified by logistic regression models fitted for 30-day and 72-hour mortality

Outcome	Model	Estimate (SE)	OR (95% CI)	P value
30-day mortality	CFS score (≥5)	0.94 (0.20)	2.55 (1.74 to 3.74)	<0.001*
	Urea (>7mmol/L)	0.92 (0.19)	2.50 (1.71 to 3.65)	<0.001*
	Consolidation	0.71 (0.24)	2.04 (1.26 to 3.29)	0.004*
	Age (≥65 years)	0.87 (0.21)	2.39 (1.58 to 3.64)	<0.001*
	FiO_2_ (>21)	0.51 (0.18)	1.67 (1.17 to 2.38)	0.005*
	Sex (male)	0.43 (0.19)	1.53 (1.06 to 2.22)	0.023
	Respiratory rate (≥30 breaths/min)	0.86 (0.22)	2.36 (1.55 to 3.61)	<0.001
72-hour mortality	CFS score (≥5)	0.96 (0.34)	2.60 (1.34 to 5.06)	0.005
	Urea (>7mmol/L)	1.25 (0.43)	3.50 (1.50 to 8.18)	0.004
	Consolidation	1.83 (0.74)	6.21 (1.45 to 26.66)	0.014
	Age (≥65 years)	0.95 (0.45)	2.59 (1.06 to 6.30)	0.036
	SpO_2_ (≤94)	0.90 (0.31)	2.46 (1.34 to 4.51)	0.004
	FiO_2_ (>21)	1.13 (0.31)	3.08 (1.67 to 5.68)	<0.001

CFS, Clinical Frailty Scale; FiO_2_, fraction of inspired oxygen; SpO_2_, oxygen saturation by pulse oximetry.

## Discussion

We analysed the accuracy with which admission CURB-65, NEWS2 and qSOFA scores predict ICU admission, early in hospital mortality and all-cause mortality within 30 days of hospital admission in the context of COVID-19. In general, calibration was poor as all three scores underestimated the risk of adverse outcomes. CURB-65 and qSOFA both performed poorly in comparison to their respective standard applications, suggesting their utility is limited in COVID-19. In contrast, NEWS2 and CURB-65 were better at predicting early death, defined here as death of <72 hours, where a NEWS2 threshold of 5 (the recommended threshold for urgent intervention) showed excellent NPV. We went on to derive two sets of parameters which, when combined on admission with COVID-19, may provide a more accurate prediction of mortality and may provide a useful basis for predictive scores to be validated in larger datasets.

CURB-65 was derived and validated to predict low, moderate and high risk of death at 30 days in patients with CAP and therefore assisted healthcare workers in deciding who required admission to the hospital. Pre-COVID validation demonstrated a score of <2 was consistently associated with low mortality rates of 0.4%–2.8% at 30 days.^6 15^ However, here in the COVID-19 setting, a CURB-65 score of <2 was associated with a mortality of 17%. In clinical practice, its utility has been expanded to support decision making around antimicrobial prescribing and escalation of care. We therefore performed an exploration of CURB-65 performance with respect to ICU admission and early mortality prediction. Our data suggest low CURB-65 scores may not support early COVID-19 discharge, but higher scores may still have value in predicting particularly poor outcomes; CURB-65 scores of ≥3 were associated with death in 60% of cases, compared with just 22% in the pre-COVID era.^5^ On that basis, high scores could prompt early escalation planning and inform discussions with patients and their families.

In the pre-COVID era, at the time of presentation to hospital with CAP, it was extremely unusual for the medical team to know the causal pathogen, and although it was well recognised that there was a range of virulence among the possible viral and bacterial causes of CAP, the data presented here confirm that SARS-CoV-2 is a highly virulent outlier. This finding has particular relevance to the evolving pandemic since, as transmission reduces, SARS-CoV-2 will become one of numerous endemic causes of CAP in many countries. It will therefore be important to recognise that this will reduce the performance of CURB-65 on undifferentiated CAP cases and makes a strong case for the implementation of rapid diagnostics to determine the aetiology of CAP.

NEWS2 has been widely implemented in English hospitals as a simple score consisting of routine physiological measurements. While it is most widely recognised as a simple tool to identify inpatients in need of urgent or emergent medical attention based on changing physiological measurements, it is also often used in the emergency department, where it has been validated in a number of syndromes, including sepsis and acute dyspnoea.^16 17^ We found that a NEWS2 score of <5 accurately identified a group of patients as a low-risk group for early mortality; however, it was less successful when 30-day mortality was considered. Our findings are based on a single measure of NEWS2 on admission, and future longitudinal work would be needed to confirm if established NEWS2 trigger thresholds remain valid for inpatients with COVID-19.

qSOFA was validated for use among hospital inpatients and emergency department admissions as a simple and accurate way to identify people with infections at higher risk of poor outcomes.^4 11 18^ Early data from China suggested those who survived COVID-19 had lower qSOFA scores, a finding replicated here.^19^ However, in our study, the median qSOFA in those who died within 30 days was <2, and mortality in this 'low-risk' qSOFA group was 32.5%. Taken together, the poor overall discriminatory ability of qSOFA and the poor diagnostic performance seen here suggests a qSOFA score on admission is not a useful prognostic tool in COVID-19. These findings are supported by a recent study which found qSOFA was low in people with COVID-19 admitted to critical care and could not reliably identify those at risk of death.^20^

The poor performance of qSOFA is interesting, given it was derived from cohorts of patients with sepsis, a syndrome defined as a ‘life-threatening organ dysfunction due to a dysregulated host response to infection’. It would be expected that many with COVID-19-associated mortality would meet that definition on the basis of respiratory failure. However, the striking difference between the physiology of bacterial sepsis and severe COVID-19 is that cardiovascular instability is rare in COVID-19.^18^ In our modelling of individual variables of CURB-65, we found that unlike the respiratory components, blood pressure was not independently associated with adverse outcome. Similarly, confusion, often a sign of haemodynamic compromise, was less relevant to outcomes in the CURB-65 score.

Blood pressure and mental status are integral components of the qSOFA score and in other contexts contribute to its ability to prognosticate; thus, the poor performance of qSOFA is explained by the limited effect of COVID-19 on these physiological parameters. These findings suggest COVID-19-associated mortality may be mediated by different mechanisms than conventional bacterial sepsis. An example of this may be the profound endothelial injury and abundant microthrombi identified in a recent postmortem study.^21^

Given the limited performance of the previously validated and widely used scores seen here, we explored whether performance could be improved by deriving new models. Using multiple logistic regression, we derived new models, CUCAF-SR and CUCA-SF, in an attempt to predict 30-day and 72-hour mortality, respectively. In keeping with the findings described earlier, markers of cardiovascular compromise were not independently associated with poorer outcomes, with markers of respiratory function, age and frailty appearing more relevant. This finding is supported by the findings of the ISARIC study, where respiratory function (respiratory rate and oxygen saturations), age and comorbidities, but not cardiovascular parameters, were important constituents of the 4C mortality score.^22^

Some limitations must be addressed. First, we only included a 2-week period, and it is possible demographics and outcomes may change across the course of the COVID-19 pandemic.^23^ Reassuringly, the characteristics and outcomes in the study population seen here are in keeping with those reported by the ISARIC study, one of the largest studies in this setting to date. For example, the median age here was 70 years compared with 72 years in the ISARIC study; 61% were male here compared with 59.9% in ISARIC; 17.1% here were admitted to critical care compared with 17% in ISARIC; and we observed 34% 30-day mortality, which is comparable to the hospital case fatality rate of 33.6% reported by ISARIC.^24 25^

A further limitation here is the inclusion only of those people admitted to hospital, thus excluding those well enough to be discharged from the emergency department. As a consequence, the observed risk presented among low-risk categories seen here may, in theory, be inflated; for example, it is possible only the sickest patients with CURB-65 scores of 0–1 were admitted. However, the main derivation dataset for the CURB-65 score included only patients admitted to the hospital, replicating the methods here.^5^ Similarly, a large validation study that included both admissions of those discharged directly from the emergency department found that 30-day mortality in those admitted to hospital with a low-risk CURB-65 score remained low at 0.0%–1.6%.^15^ This supports the conclusion that the observed high mortality among low CURB-65 scores in this study was due to SARS-CoV-2 virulence rather than study design. Conversely, some patients presented to the emergency department in a moribund state and did not survive long enough for a viral swab to be taken. Such patients were not included in our study, and generalisability to that small subset of patients may be limited. Data collection here did not include assessment of detailed patient demographics or comorbidities, but instead focused on clinical measurements normally taken at presentation to the hospital. Characteristics such as obesity, ethnicity and comorbidities are reported to be relevant to COVID-19 outcomes but are not included here.^26 27^ It may be that a combination of clinical parameters and patient characteristics is more informative than either in isolation and validation of such an approach is required.^28^ We defined ‘early mortality’ as death occurring within 72 hours of admission in order to capture patients who deteriorated quickly, but within a time frame that would allow them to have a known SARS-CoV-2 status and be identified by our investigators. This 72-hour timepoint differs from the 24-hour timepoint used in the NEWS2 score’s validation studies but, given the above constraints, was considered a pragmatic approach for our analysis. Finally, ICU admission is not appropriate for all patients, and our analysis of ICU admissions may be prone to unmeasured confounding in that regard.

The strengths of this study lie in the prospective collection of data on consecutive admissions from multiple regional hospitals with rigorous assessment of the performance of each score. These readily available data were compiled from real-world clinical assessment, and outcomes followed usual clinical care. We also demonstrate the hitherto underappreciated potential of highly trained and motivated specialty trainees and their ability to coordinate and collaborate for research.

## Conclusion

CURB-65, NEWS2 and qSOFA underestimate 30-day mortality among patients admitted to the hospital with COVID-19. CURB-65 and NEWS2 were slightly better at predicting early mortality. However, our data suggest CURB-65 should not be used to prognosticate in the setting of COVID-19 pneumonia since low CURB-65 scores were associated with high mortality rates. We provide a set of clinical parameters which appear relevant to outcomes in COVID-19 and should be considered in future studies aimed at deriving COVID-19-specific prognostic tools.
