# Supplementary material for: Utility of established prognostic scores in COVID-19 hospital admissions: multicentre prospective evaluation of CURB-65, NEWS2 and qSOFA
Source: BMJ Open Respir Res. 2020 Dec 7;7(1):e000729. doi: 10.1136/bmjresp-2020-000729 (PMC7722817; doi:10.1136/bmjresp-2020-000729)
Supplement: Supplementary data [file bmjresp-2020-000729supp001.pdf]

Supplementary data and figures:

List of participating organisations:

Blackpool Teaching Hospitals NHS Foundation Trust, Blackpool, UK

Countess of Chester Hospital NHS Foundation Trust, Chester, UK

Liverpool University Hospitals NHS Foundation Trust, Liverpool, UK

Manchester University NHS Foundation Trust, Manchester, UK

Mid Cheshire Hospitals NHS Foundation Trust, Leighton, UK

Lancashire Teaching Hospitals NHS Foundation Trust, Preston, UK

Southport and Ormskirk Hospital NHS Trust, Southport, UK

Liverpool Heart & Chest Hospital NHS Foundation Trust, Liverpool, UK*

*=No acute admissions, outcomes recorded from critical care transfers only.

Table S1: Demographics and clinical characteristics at presentation with comparison by 72-hour outcome

	**Missing**	**Death within 72 hours**		
		Alive	Dead	p
**n**		767	63	
**Age (Years)**	1 (0.1%)	69 (58,79)	80 (75,87)	<0.001
**Sex (Male)**	0 (0.0%)	474 (61.8%)	35 (55.6%)	0.348
**Clinical Frailty Score**	81 (9.8%)	3 (2,6)	6 (4,7)	<0.001
**Temp (°C)**	3 (0.4%)	37.5 (36.8,38.2)	37.5 (37.0,38.4)	0.356
**Respiratory rate(/min)**	2 (0.2%)	23 (20,28)	26 (22,34)	<0.001
**Heart rate (bpm)**	1 (0.1%)	94 (81,108)	96 (82,119)	0.155
**Systolic BP (mmHg)**	2 (0.2%)	130 (115,145)	130 (109,151)	0.721
**Diastolic BP (mmHg)**	2 (0.2%)	75 (66,84)	70 (62,85)	0.162
**SpO2**	1 (0.1%)	94 (90,96)	92 (86,94.5)	0.002
**Supplemental oxygen**	2 (0.2%)	297 (38.7%)	43 (68.3%)	<0.001
**SpO2/FiO2 ratio**	2(0.2%)	419 (313, 448)	209 (116, 410)	<0.001
**Confusion**	0 (0.0%)	159 (20.7%)	24 (38.1%)	0.002
**Urea (mmol/L)**	8 (1.0%)	7.0(4.9,10.8)	12.3(8.9,19.9)	<0.001
**Consolidation**	4 (0.5%)	624 (81.7%)	60 (96.8%)	0.001
**CRP (mg/L)**	10 (1.2%)	100.0(46.0,165.2)	169.0(92.0,244.0)	<0.001
**WCC 10^9/L)**	4 (0.5%)	7.3(5.5,10.0)	8.2(6.6,11.6)	0.014
**Neutrophils (10^9/L)**	7 (0.8%)	5.7(3.8,8.3)	7.2(4.6,9.9)	0.014
**Lymphocytes (10^9/L)**	9 (1.1%)	0.8(0.6,1.1)	0.7(0.4,1.0)	0.01
**NLR**	9 (1.1%)	159.5(96.0,202.0)	120.0(39.0,179.5)	0.003

Abbreviations: CFS=Clinical frailty scale; BP=blood pressure; SpO2=oxygen saturation by pulse oximetry; FiO2=fraction of inspired oxygen; CRP=C-reactive protein; WCC=White cell count; NLR=Neutrophil/Lymphocyte ratio

Table S2: Restricted analysis of CURB65 0-1.

Baseline demographics and clinical characteristics with comparison by outcome at 30 days. Data are presented as n(%) or median (IQR). P-values are calculated using Fisher's exact test for categorical variables, Mann-Whitney (M) test for continuous variables since none of the continuous variables follow the assumption of normality (Shapiro normality test p-value=<0.001 for all of them).

	**Missing**	**All**	**Death by 30 days**		
			Alive	Dead	p
**n**		317	261	56	
**Characteristics at presentation**					
Age (Years)	0 (0.0%)	58.0 (50.0,64.0)	57.0 (49.0,63.0)	61.0 (57.8,66.3)	<0.001
Sex (Male)	0 (0.0%)	199 (62.8%)	164 (62.8%)	35 (62.5%)	1.000
CFS	38 (12%)	2 (1,3)	2 (1,3)	3 (1,5)	0.014
Temp (°C)	2 (0.6%)	37.6 (37.0,38.2)	37.6 (37.0,38.2)	37.7 (37.1,38.2)	0.638
Respiratory rate (/min)	1 (0.3%)	22.0 (20.0,26.0)	22.0 (20.0,26.0)	24.0 (20.0,27.3)	0.336
Heart rate (bpm)	0 (0.0%)	96 (84,109)	97 (84,109)	95 (82,113)	0.744
Systolic BP (mmHg)	0 (0.0%)	132 (119,147)	133 (120,148)	132 (118,144)	0.486
Diastolic BP (mmHg)	0 (0.0%)	78.0 (70.0,86.0)	79.0 (72.0,87.0)	72.5 (66.0,84.0)	0.005
SpO2	0 (0.0%)	94.0 (91.0,96.0)	94.0 (91.0,96.0)	93.5 (90.0,96.0)	0.649
Supplemental oxygen	1 (0.3%)	113 (18.7%)	86 (33.0%)	27 (48.2%)	0.044
SpO2/FiO2 ratio	1(0.3%)	427 (320, 448)	433 (343,448)	358 (158,443)	0.005
Confusion	0 (0.0%)	8 (2.5%)	7 (2.7%)	1 (1.8%)	1.000
**Investigations**					
Urea (mmol/L)	4 (1.3%)	5.0(4.0,6.5)	4.9(3.9,6.3)	6.1(4.3,7.8)	0.005
CRP (mg/L)	3 (0.9%)	109.0(51.0,168.8)	102.0(48.5,161.5)	136.0(87.5,180.0)	0.027
WCC (10^9/L)	2 (0.6%)	7.2(5.4,9.2)	7.1(5.4,9.0)	7.6(5.5,11.3)	0.328
Neutrophils (10^9/L)	4 (1.3%)	5.4(3.7,7.7)	5.4(3.7,7.5)	5.8(4.2,8.9)	0.261
Lymphocytes (10^9/L)	4 (1.3%)	0.9(0.6,1.2)	0.9(0.7,1.2)	0.7(0.5,1.1)	0.008
NLR	4 (1.3%)	169.0(98.0,201.0)	169.0(99.5,201.5)	151.5(78.0,199.2)	0.595

Abbreviations: CFS=Clinical frailty scale; BP=blood pressure; SpO2=oxygen saturation by pulse oximetry; FiO2=fraction of inspired oxygen; CRP=C-reactive protein; WCC=White cell count; NLR=Neutrophil/Lymphocyte ratio

Table S3: Score performance for ICU admission as outcome (sensitivity, specificity, PPV, NPV)

			ICU admission			
**Tool**	**Score(n)**	**Number of ICU admission (%)**	**Sensitivity**	**Specificity**	**PPV**	**NPV**
CURB65 (n=605) +	<2 (273)	68 (24.9%)				
	≥2 (332)	42 (12.7%)	0.38	0.41	0.13	0.75
	<3 (434)	94 (21.7%)				
	≥3 (171)	16 (9.4%)	0.15	0.69	0.09	0.78
NEWS2 (n=730)	<5 (215)	21 (9.8%)				
	≥5 (515)	95 (18.4%)	0.82	0.32	0.18	0.90
qSOFA (n=730)	<2 (596)	103 (17.3%)				
	≥2 (134)	13 (9.7%)	0.11	0.80	0.10	0.83

Abbreviations: PPV = Positive predictive value; NPV = Negative predictive value
+=Analysis restricted to those with consolidation on chest radiograph.

Table S4: Logistic regression models for 30-day mortality fitted using the individual constituents of each score.

Model	Variables	OR (95%CI)	p-value
**CURB-65**	Confusion (Yes)	1.29 (0.83 - 1.99)	0.258
**(n=605)** +	Urea (>7mmol/L)	2.94 (1.97 - 4.38)	<0.001*
	Respiratory Rate (≥30)	2.37 (1.52 - 3.69)	<0.001*
	BP (SBP<90 or DBP≤60)	1.42 (0.88 - 2.31)	0.155
	Age (≥ 65)	3.25 (2.13 - 4.95)	<0.001*
**NEWS2**	Respiratory Rate		
**(n=730)**	2 points	1.29 (0.84 – 1.97)	0.245
	3 points	1.90 (1.25 – 2.90)	0.003*
	SpO2		
	1 point	0.81 (0.50 – 1.30)	0.379
	2 points	1.59 (0.97 – 2.60)	0.064
	3 points	1.47 (0.99 – 2.20)	0.058
	FiO2		
	2 point	2.00 (1.43 – 2.80)	<0.001*
	Temperature		
	1 point	1.43 (0.99 – 2.06)	0.059
	2 points	1.68 (0.90 – 3.14)	0.102
	3 points	1.33 (0.32 – 5.42)	0.694
	Systolic BP		
	1 point	1.19 (0.68 – 2.06)	0.541
	2 points	2.06 (1.04 – 4.06)	0.038*
	3 points	1.23 (0.56 – 2.70)	0.606
	Heart rate		
	1 point	0.65 (0.45 – 0.95)	0.026*
	2 points	0.78 (0.49 – 1.25)	0.303
	3 points	0.84 (0.39 – 1.79)	0.651
	Confusion (Yes)	2.35 (1.63 – 3.40)	<0.001*
**qSOFA**	Respiratory Rate (≥ 22)	1.85 (1.33 - 2.58)	<0.001*
**(n=730**	Systolic BP (≤100)	1.74 (1.04 - 2.91)	0.035*
	Altered mental status (Yes)	2.37 (1.66 - 3.38)	<0.001*

+=Analysis restricted to those with consolidation on chest radiograph.
